# Complementing Bone Mineral Density Assessment: DXA-Based Radiomics for Vertebral Evaluation in Older Adults

**DOI:** 10.3390/medicina62101989

**Published:** 2026-10-15

**Authors:** Yeny Concha-Cisternas, Eduardo Guzmán-Muñoz, Dario Barrera-González, Walter Sepúlveda-Loyola, Lincoyán Fernández-Huerta, Igor Cigarroa-Cuevas, Rodrigo Yañez-Sepúlveda, Felipe Montalva-Valenzuela, Exal Garcia-Carrillo, Iván Molina-Marquez, Júlio Brugnara Mello

**Affiliations:** 1 Escuela de Kinesiología, Facultad de Salud, Universidad Santo Tomás, Talca 3460000, Chile; eguzmanm@santotomas.cl; 2 Vicerrectoría de Investigación e Innovación, Universidad Arturo Prat, Iquique 1100000, Chile; 3 Escuela de Kinesiología, Facultad de Ciencias de la Salud, Universidad Autónoma de Chile, Talca 3460000, Chile; 4 Faculty of Health and Social Sciences, Universidad de Las Américas, Santiago 7500975, Chile; wsepulveda@udla.cl; 5 Escuela de Kinesiología, Facultad de Ciencias de la Rehabilitación y Calidad de Vida, Universidad San Sebastián, Concepción 4030000, Chile; lincoyan.fernandez@uss.cl; 6 Escuela de Kinesiología y Terapia Ocupacional, Facultad de Ciencias de la Salud, Universidad Católica Silva Henríquez, Santiago 8320000, Chile; icigarroac@ucsh.cl; 7 Faculty of Education and Social Sciences, Universidad Andrés Bello, Viña del Mar 2200055, Chile; rodrigo.yanez.s@unab.cl; 8 School of Medicine, Universidad Espíritu Santo, Samborondón 092301, Ecuador; 9 Escuela de Entrenador en Actividad Física y Deporte, Facultad de Ciencias Humanas, Universidad Bernardo O’Higgins, Santiago 8370040, Chile; felipe.montalva@ubo.cl; 10 Department of Physical Activity Sciences, Faculty of Education Sciences, Universidad Católica del Maule, Talca 3480112, Chile; exal.garcia@gmail.com; 11 Department of Physical Activity Sciences, Universidad de Los Lagos, Osorno 5290000, Chile; 12 Faculty of Education, Pedagogy in Physical Education, Universidad Adventista de Chile, Chillán 3780000, Chile; ivanmolina@unach.cl or; 13 Doctoral Program in Physical Activity Sciences, Universidad Católica del Maule, Talca 34809112, Chile; 14 Research Group on Musculoskeletal Adaptations to Sports Training, Escuela de Educación Física, Pontificia Universidad Católica de Valparaíso, Valparaíso 2362807, Chile; julio.mello@pucv.cl

**Keywords:** osteoporosis, DXA, radiomics, texture analysis, bone mineral density

## Abstract

*Background and Objectives*: Dual-energy X-ray absorptiometry (DXA) provides areal bone mineral density (BMD) and T-scores but does not fully characterize image texture. This study examined the relationship between lumbar T-score and DXA-derived Shannon entropy, a first-order descriptor of image-intensity heterogeneity, and whether entropy differed across densitometric categories in older adults. *Materials and Methods*: This cross-sectional study included 271 community-dwelling adults aged ≥60 years who underwent lumbar DXA. Regions of interest from L1–L4 were segmented, gray levels were quantized to 8 bits, and entropy was calculated for each evaluable vertebra and averaged at the participant level. Pearson correlation, one-way ANOVA with Tukey post hoc testing, effect sizes, tertile-based discordance analysis, and multiple linear regression were applied. *Results*: T-score and entropy showed a weak positive correlation (r = 0.203; R^2^ = 0.04; *p* < 0.001). Entropy differed across densitometric categories (F = 9.04; *p* < 0.001; η^2^ = 0.063). Values were similar in the normal and osteopenic groups (2.151 ± 0.063 vs. 2.150 ± 0.063), whereas the osteoporosis group showed lower entropy (2.111 ± 0.076; *p* < 0.001). Low entropy was also observed in 27.5% of participants with normal BMD and 29.0% with osteopenia. After adjustment for age, sex, and BMI, T-score was not independently associated with entropy (*p* = 0.127). *Conclusions*: DXA-derived Shannon entropy showed limited correspondence with lumbar T-score and lower values in osteoporosis. It should be interpreted as a descriptor of DXA image-intensity heterogeneity, not as a direct measure of trabecular microarchitecture. Further validation is required before clinical application.

## 1. Introduction

Osteoporosis is a systemic skeletal disease characterized by decreased bone strength, resulting from the interaction between low bone mineral density (BMD) and alterations in bone tissue quality, including deterioration of its microarchitecture [1]. This condition increases the risk of fragility fractures, particularly in older adults and postmenopausal women [1,2]. In the context of population aging, osteoporosis constitutes a major public health problem, because fragility fractures are associated with increased morbidity and mortality, functional decline, loss of independence, reduced quality of life, and high healthcare costs [3,4,5]. Despite the availability of effective interventions to prevent these fractures, osteoporosis remains a frequently underdiagnosed and undertreated disease [6]. Therefore, the timely detection of alterations in bone health is essential to identify individuals at higher risk and to guide preventive, diagnostic, and therapeutic decisions.

Bone health is primarily assessed using dual-energy X-ray absorptiometry (DXA), which quantifies BMD and calculates the T-score, the standard desitometric index used in routine clinical practice [7]. Based on the World Health Organization criteria, the T-score enables the classification of individuals as having normal bone density, osteopenia, or osteoporosis [8]. DXA is widely used in clinical practice owing to its availability, low radiation exposure, adequate reproducibility, and ability to estimate fracture risk [9,10]. However, despite these advantages, the information provided by DXA may be insufficient to comprehensively characterize bone strength, as this depends not only on mineral content but also on bone geometry, cortical and trabecular distribution, and tissue microarchitecture [11,12]. This limitation is important because the strength of the trabecular compartment is determined by trabecular number, thickness, separation, connectivity, orientation, and spatial distribution [10]. Consequently, two vertebrae with identical BMD may differ substantially in their internal architecture and in their mechanical competence.

Therefore, it is necessary to explore complementary tools that provide additional information from densitometric images, without replacing the clinical utility of DXA but rather expanding its capacity to characterize bone tissue [12]. In this context, radiomics is a computational approach aimed at the systematic extraction of quantitative features from medical images [13]. Through the analysis of previously delineated regions of interest, it transforms intensity and texture patterns into objective numerical variables, potentially associated with the heterogeneity and organization of the underlying tissue [14]. Unlike conventional densitometric measurements, which summarize the mineral quantity in a single global value, radiomics analyzes the distribution of gray levels and their variations within the image [13].

In the musculoskeletal field, several studies have demonstrated that radiomic features derived from radiographs, computed tomography, and DXA provide complementary information on bone quality and improve the discrimination of osteoporosis, skeletal fragility, and fracture risk beyond that achieved by BMD alone [15,16,17]. Among first-order radiomic descriptors, Shannon entropy quantifies the degree of heterogeneity in the distribution of gray levels within a region of interest, where high values indicate greater intensity variability and low values represent a more homogeneous texture [18,19]. Because changes in bone tissue can modify the gray-level patterns represented in medical images, Shannon entropy may capture variations in image heterogeneity related to skeletal status. However, as a first-order histogram-based feature, it reflects the distribution of image intensities rather than the spatial organization of trabecular structures and therefore should not be interpreted as a direct measure of trabecular microarchitecture [20]. Thus, Shannon entropy could complement the information provided by BMD and the T-score by capturing variations in DXA image-intensity heterogeneity that are not summarized by mineral density alone.

Based on this background, the present study aimed to analyze the relationship between the lumbar T-score and DXA-derived Shannon entropy in older adults, and to determine whether this descriptor differs across the densitometric categories of normal bone density, osteopenia, and osteoporosis. It was hypothesized that Shannon entropy would show an association with the lumbar T-score and lower values in participants with osteoporosis, which would support its potential as a complementary descriptor of vertebral DXA image-texture heterogeneity.

## 2. Materials and Methods

### 2.1. Study Design and Participants

An observational, analytical, cross-sectional study was conducted. The study population consisted of older adults over 60 years of age belonging to community clubs and centers in the Maule Region, Chile. The final sample comprised 271 participants, selected through non-probability convenience sampling. The sample size was estimated a priori based on the primary objective of examining the association between lumbar T-score and DXA-derived Shannon entropy. Previous studies have demonstrated the applicability of radiomic texture analysis for the classification of bone mineral loss and the differentiation of densitometric categories [21], while associations between vertebral texture features, bone mineral density, and trabecular microarchitecture have also been reported using other imaging modalities [22]. However, because these studies differed from the present investigation in terms of imaging modality, radiomic feature definition, image processing, and statistical approach, a conservative minimum correlation of r = 0.20 was assumed. Considering a two-sided significance level of 0.05 and a statistical power of 90%, a minimum of 259 evaluable participants was required. The final analytical sample comprised 271 participants, exceeding the minimum estimated sample size. Individuals aged 60 years or older, of both sexes, belonging to community organizations for older adults, and able to understand and perform simple instructions—a condition verified through the Abbreviated Mini-Mental State Examination—were included [23]. In addition, participants were required to be functionally independent according to the Functional Examination of the Older Adult used in Chile [24], and to voluntarily agree to participate by signing an informed consent form.

Participants presenting conditions capable of interfering with the densitometric measurement or the image analysis were excluded, including the presence of metallic artifacts in the lumbar region, residual contrast material, or extensive aortic calcifications overlapping the measurement field [25]. Participants with moderate or severe vertebral compression fractures, significant vertebral deformities, or advanced degenerative changes—such as prominent osteophytes or severe facet sclerosis—were also excluded when these conditions could alter the estimation of BMD or distort vertebral texture patterns. Additionally, subjects with metabolic bone diseases other than osteoporosis, neoplastic involvement of the vertebral region, or a history of pharmacological treatments affecting bone metabolism were excluded, insofar as this information was available. Finally, images with insufficient resolution, loss of digital information, or the impossibility of adequately segmenting the region of interest were discarded [25]. The study was conducted in accordance with the ethical principles for research involving human participants. The research protocol was approved by the Scientific Ethics Committee of a Chilean university (Approval No. 26-46). All participants were informed about the objectives and procedures of the study before the evaluation.

### 2.2. Sociodemographic and Anthropometric Outcomes

Sociodemographic and anthropometric characterization variables were recorded, including age, sex, body weight, height, body mass index, and waist circumference. Body weight was measured with a calibrated electronic scale (Seca, Hamburg, Germany), with participants barefoot and wearing light clothing [26]. Height was determined using a portable stadiometer (Seca 274, Seca AG, Reinach, Switzerland), keeping the participant in an upright position and following standardized anthropometric procedures. Body mass index (BMI) was calculated as body weight in kilograms divided by height in meters squared, expressed in kg/m^2^. Finally, waist circumference was measured using a flexible, inextensible measuring tape (Seca 201, Reinach, Switzerland), recording the value at the end of a normal expiration, at the midpoint between the lower costal margin and the iliac crest [27].

### 2.3. Densitometric Assessment by DXA

Bone mineral density was assessed by DXA using a GE Lunar Prodigy scanner (GE Lunar Healthcare, Madison, MI, USA) and enCORE software version 13.60. The equipment was calibrated daily using a standard phantom block to ensure the stability of the measurements. All assessments were performed under standardized conditions in the laboratory of a Chilean university. Participants were positioned supine on the examination table, with the lower limbs resting on a standard padded support that kept the hips and knees in approximately 90° of flexion [25]. This position was used to reduce physiological lumbar lordosis, promote adequate alignment of the lumbar spine, and minimize possible artifacts during image acquisition. Anteroposterior projection images of the lumbar spine were obtained, considering vertebrae L1–L4. The images were subsequently reviewed to verify adequate anatomical delineation and the absence of relevant artifacts that could compromise the densitometric interpretation or the texture analysis. Bone mineral density was expressed in g/cm^2^ and as a T-score, calculated relative to a young adult reference population of the same sex. Diagnostic classification was performed according to conventional densitometric criteria: normal when T-score ≥ −1.0, osteopenia when −2.5 < T-score < −1.0, and osteoporosis when T-score ≤ −2.5 [8].

### 2.4. Image Analysis, ROI Segmentation, and Radiomic Processing

The digital lumbar spine images obtained by DXA underwent a preparation process prior to the calculation of Shannon entropy. The images used for texture analysis were obtained directly from the DXA system after completion of the standard acquisition and analysis procedure. No additional filtering, smoothing, resampling, or intensity normalization was applied. The preparation process was therefore limited to standardized delineation of the anatomical region of interest and subsequent gray-level quantization. The analysis was performed on anteroposterior projection images, considering the evaluable lumbar vertebrae from L1 to L4 [20]. For each evaluable vertebra, a region of interest (ROI) corresponding to the vertebral body was delineated using a supervised semiautomatic procedure [13]. The ROIs for the entire study sample were initially delineated by the principal investigator. To assess inter-observer reproducibility, a random subsample of 30 participants was subsequently selected, and the corresponding images were independently reanalyzed by a second investigator who was blinded to the ROI delineations and entropy values obtained by the principal investigator. The second investigator independently delineated the ROIs of all evaluable vertebrae from L1 to L4 using the same segmentation and image-processing protocol applied in the primary analysis. The resulting participant-level entropy values were then compared with those originally obtained by the principal investigator to assess inter-observer reproducibility. Shannon entropy was calculated separately for each evaluable vertebra and averaged at the participant level, consistent with the primary analysis. The purpose of this segmentation was to isolate the region corresponding to the trabecular compartment and to reduce the influence of structures not representative of the tissue of interest. Subsequently, the segmented images underwent gray-level quantization to 8 bits, corresponding to 256 discrete intensity levels. This quantization level was selected to preserve relatively fine gray-level information while providing a uniform discrete intensity scale for radiomic analysis, consistent with previously reported radiomics standardization approaches [20,28]. Importantly, the use of 256 levels was not intended to represent an optimal or biologically determined threshold, but rather a predefined image-processing parameter applied consistently across all participants. Entropy values were interpreted as quantitative descriptors of DXA image-intensity heterogeneity and not as direct measurements of three-dimensional trabecular microarchitecture [29].

### 2.5. Calculation of Shannon Entropy

The central radiomic biomarker was Shannon entropy, calculated from the distribution of gray levels within each segmented region of interest. For each ROI, the proportion of pixels corresponding to each intensity level present in the image was estimated, generating a probability distribution of the gray levels [19]. Shannon entropy was calculated using the following expression:

*H* = −Σ_i__=1_
*p_i_ log*_2_(*p_i_*)

where *H* is the Shannon entropy, *pi* represents the probability of occurrence of gray level i within the ROI, and the summation is performed over all gray levels present in the image. Quantization was performed at 8 bits, corresponding to a maximum of 256 discrete gray levels; consequently, entropy was expressed in bits.

**Figure 1:**
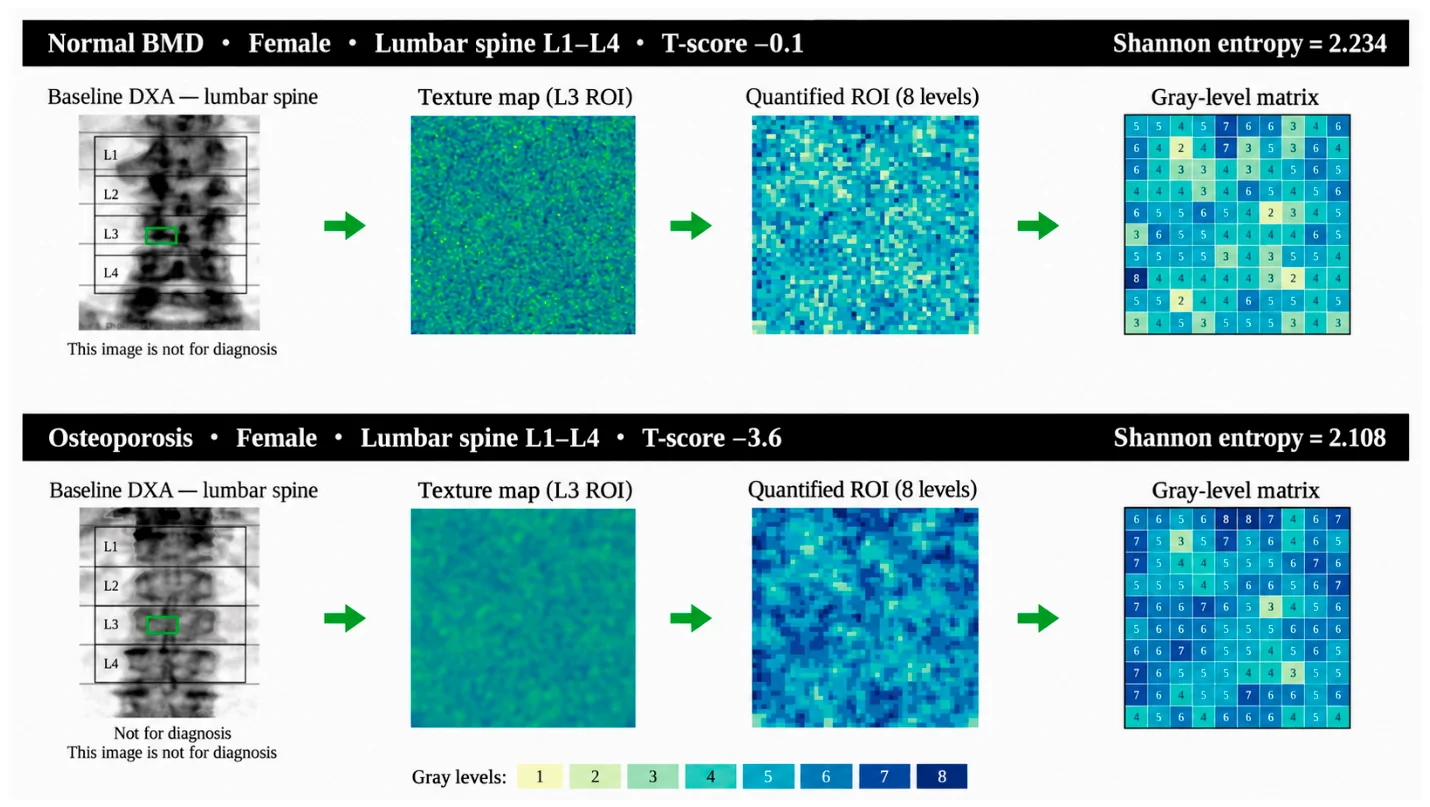
Visual examples of the DXA-based Shannon-entropy texture pipeline in two representative women with contrasting bone status: normal bone mineral density (**top**) and osteoporosis (**bottom**). For each case, the trabecular region of interest (green box) within a lumbar vertebral body on the baseline DXA scan is mapped to gray levels, quantized, and summarized by its Shannon entropy. The osteoporotic case shows a more homogeneous texture and lower entropy than the normal case, consistent with the group-level findings. Columns from left to right: baseline DXA image with the ROI; gray-level texture map; schematic quantized ROI; and schematic gray-level matrix. The eight-level quantized ROI is presented for illustrative purposes only; Shannon entropy was calculated using 8-bit quantization corresponding to 256 gray levels. DXA = dual-energy X-ray absorptiometry; ROI = region of interest; L1–L4 = first to fourth lumbar vertebrae.

Entropy quantifies the heterogeneity in the distribution of intensities within a region of interest. High values indicate greater diversity of gray levels and, therefore, a more heterogeneous texture; conversely, low values reflect a more uniform distribution of intensities [30]. The entropy value was calculated for each evaluable vertebra and subsequently averaged at the individual level, yielding a continuous measure of DXA image-texture heterogeneity per participant. The processing workflow—from the baseline DXA image and delineation of the trabecular ROI to the texture map, gray-level quantization, and finally the entropy value—is illustrated in Figure 1 for two representative participants with normal BMD and osteoporosis, respectively.

### 2.6. Statistical Analysis

Data processing, cleaning, and analysis were performed using Python version 3.10 in the Google Colab environment. Standard scientific libraries were used, including Pandas version 1.5.3 for data management and organization, SciPy version 1.10 for inferential statistical analyses, and Statsmodels version 0.13.5 for statistical modeling. The entropy values, derived from the image analysis, and the densitometric and anthropometric variables, recorded in separate databases, were integrated at the individual level through matching by nominal identification. Continuous quantitative variables, including age, BMI, T-score, and Shannon entropy, were described using the mean, standard deviation, and 95% confidence interval. The normality of the variables was assessed using the Kolmogorov–Smirnov test and the homogeneity of variances using the Levene test.

To compare mean Shannon entropy values across the densitometric categories of normal bone density, osteopenia, and osteoporosis, a one-way analysis of variance was performed. The overall magnitude of the differences was estimated using eta squared (η^2^). When statistically significant differences were identified, Tukey’s post hoc test was applied for multiple comparisons between groups. The magnitude of pairwise differences was estimated using Cohen’s d, with values of approximately 0.2, 0.5, and 0.8 interpreted as small, moderate, and large effects, respectively. The relationship between lumbar T-score and Shannon entropy was subsequently assessed using Pearson’s correlation coefficient. The squared correlation coefficient (r^2^) was calculated as a descriptive measure of the proportion of variance linearly shared between the two variables.

For exploratory purposes, Shannon entropy was stratified into tertiles, with the lower tertile defined as the range of lowest texture heterogeneity within the sample. This categorization was used to examine the distribution of participants with lower entropy across the densitometric categories, without establishing clinical cutoff points. Finally, a multiple linear regression model was fitted to determine whether the association between lumbar T-score and Shannon entropy persisted after accounting for age, sex, and body mass index. Shannon entropy was entered as the dependent variable, whereas lumbar T-score, age, sex, and body mass index were included as predictor variables. The overall significance of the model was assessed using the F statistic, and its explanatory capacity was evaluated using R^2^ and adjusted R^2^. Inter-observer reproducibility of participant-level Shannon entropy was assessed in a random subsample of 30 participants using a two-way random-effects intraclass correlation coefficient for absolute agreement based on single measurements [ICC(2,1)]. The ICC was reported with its 95% confidence interval. Statistical significance was *p* < 0.05.

## 3. Results

### 3.1. Sample Characteristics

The cohort included 271 participants, predominantly women (81.5%), with a mean age of 72.5 ± 8.3 years and a BMI of 29.8 ± 5.0 kg/m^2^. On average, women had lower BMD than men (T-score: −1.86 ± 2.40 vs. −0.34 ± 2.40) and, in parallel, slightly lower entropy (2.134 ± 0.07 vs. 2.171 ± 0.07), pointing to somewhat lower DXA image-texture heterogeneity in women. The complete characteristics are summarized in Table 1.

### 3.2. Entropy According to Densitometric Category

**Figure 2:**
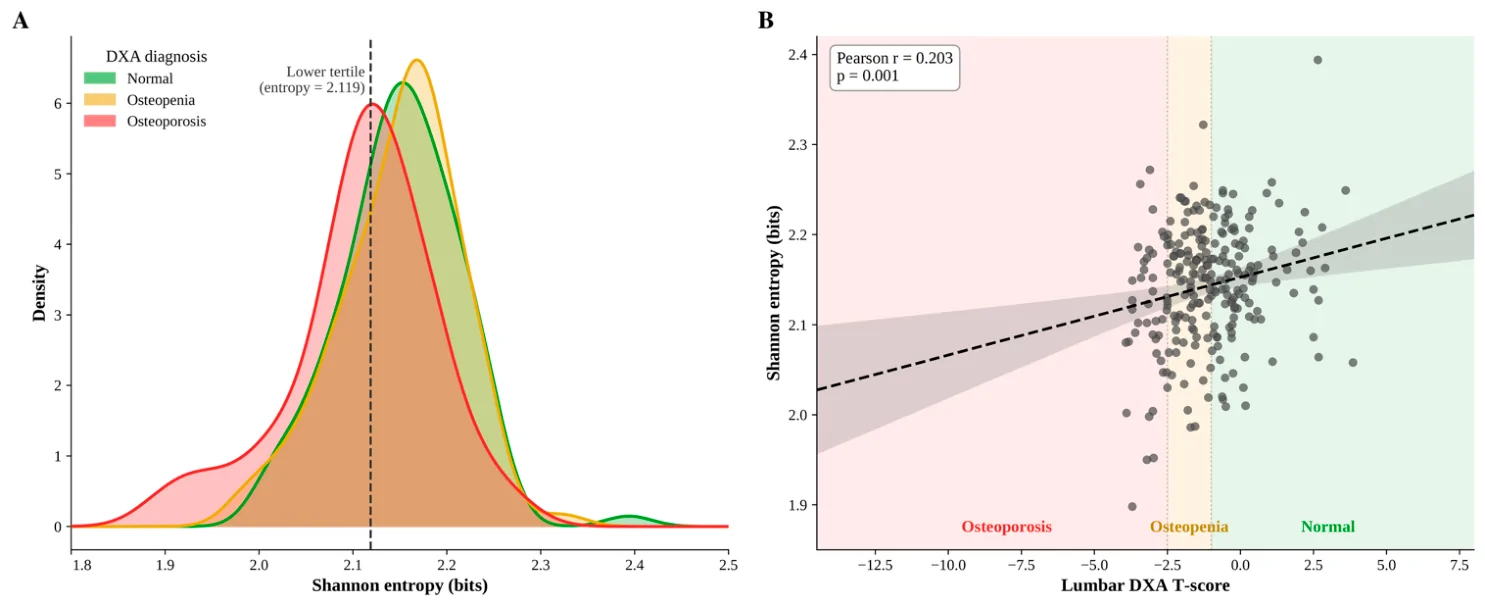
Relationship between lumbar DXA T-score and DXA-derived Shannon entropy. (**A**) Kernel density estimates of Shannon entropy across densitometric categories. The normal and osteopenia distributions largely overlap, whereas the osteoporosis distribution is shifted toward lower entropy values. The vertical dashed line indicates the sample-specific cutoff for the lower tertile of entropy. (**B**) Scatter plot of lumbar T-score and Shannon entropy with the fitted linear regression line and 95% confidence interval (*r* = 0.203; *p* < 0.001; R^2^ = 0.04). Background shading represents the densitometric categories of osteoporosis, osteopenia, and normal bone density.

Mean entropy was compared among the three densitometric groups (Table 2). Women represented 68.8% (75/109) of the normal BMD group, 90.3% (84/93) of the osteopenia group, and 89.9% (62/69) of the osteoporosis group. The analysis of variance showed significant overall differences (F = 9.04; *p* < 0.001), although of modest magnitude, with the densitometric category explaining approximately 6% of the variation in entropy (η^2^ = 0.063). Entropy did not decrease progressively as bone density worsened. The normal and osteopenic groups had virtually identical values (2.151 ± 0.063 vs. 2.150 ± 0.063), with a difference below 0.1% and a null effect size (d = 0.02; *p* > 0.99); the transition from normal to osteopenia was not accompanied by any change in texture. The reduction appeared only in the osteoporosis group (2.111 ± 0.076), 1.86% lower than the normal group (*p* < 0.001), with a moderate effect size (d = 0.59). In other words, the reduction in DXA image-texture heterogeneity detected by this method was observed only in the osteoporosis group. The same behavior is observed in Figure 2A, where the curves of the normal and osteopenic groups overlap almost completely, whereas only that of the osteoporosis group shifts toward lower entropy values.

### 3.3. Relationship Between Bone Mineral Density and Entropy at the Individual Level

The lumbar T-score and Shannon entropy were correlated at the individual level (Figure 2B). The correlation was positive and statistically significant (r = 0.203; *p* < 0.001). The coefficient of determination was R^2^ = 0.04, indicating that the T-score accounted for 4% of the variance in entropy. Entropy values spanned a similar range across the densitometric spectrum, and participants with comparable T-scores showed a wide range of entropy values.

**Figure 3:**
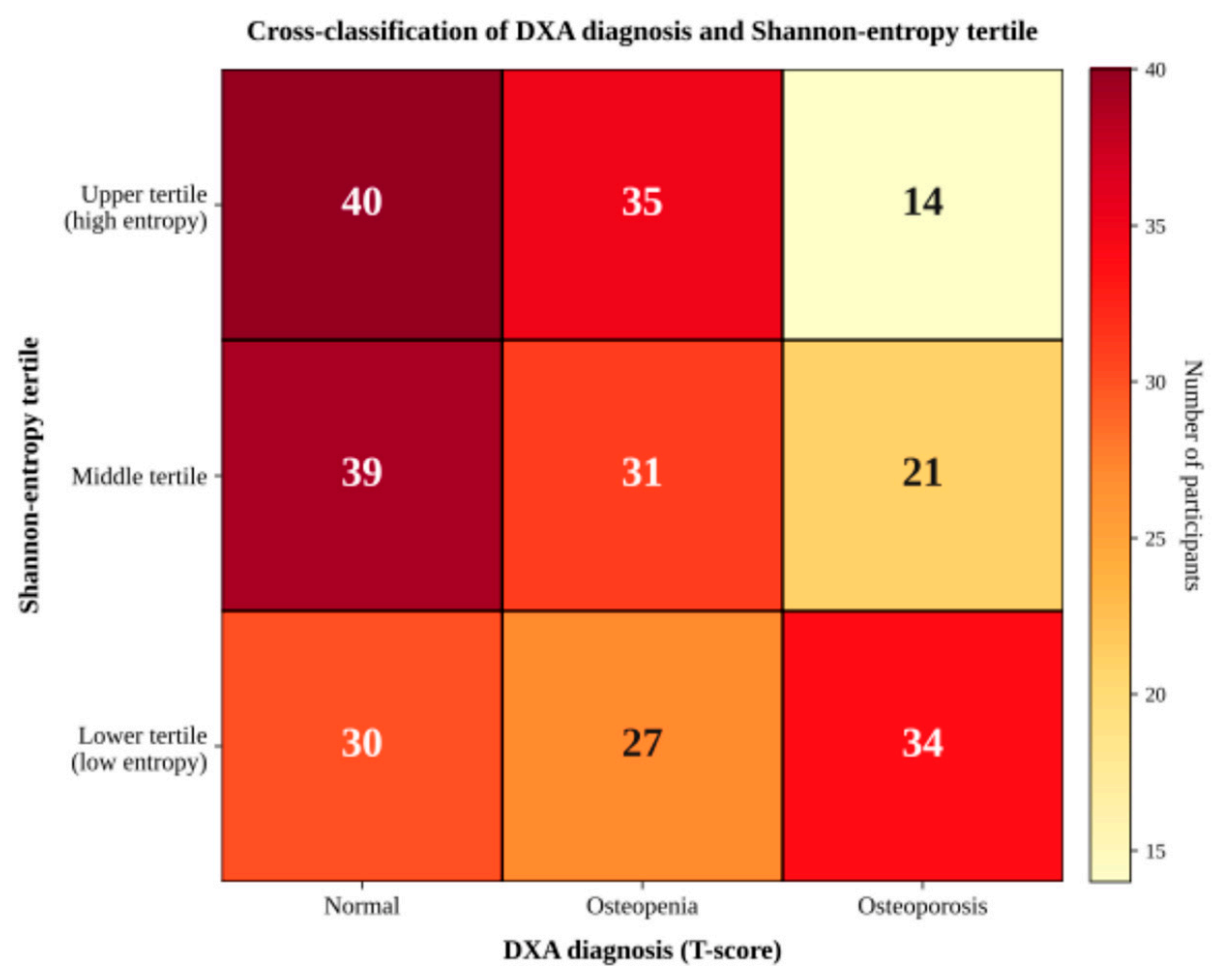
Cross-classification of densitometric diagnosis and Shannon entropy tertiles. The heatmap shows the number of male and female participants across lumbar T-score categories (normal bone density, osteopenia, and osteoporosis) and sample-specific Shannon entropy tertiles (upper, middle, and lower). Values within the cells represent participant counts. Participants of both sexes with normal bone density or osteopenia were also represented in the lower entropy tertile.

To examine the concordance between the two approaches in each person, entropy was divided into tertiles (high, medium, and low heterogeneity) and cross-tabulated with the densitometric category (Figure 3). In the osteoporosis group, most participants were in the low entropy tertile (n = 34), compared with 21 in the middle tertile and 14 in the high tertile. However, an appreciable proportion of participants with normal or osteopenic density also showed low entropy: 30 of 109 individuals with normal density (27.5%) and 27 of 93 with osteopenia (29.0%). Therefore, nearly one in four participants without a densitometric diagnosis of osteoporosis showed a uniform vertebral texture similar to that of the osteoporotic group. This discordance indicates that BMD-based classification and texture-heterogeneity-based classification do not identify exactly the same individuals.

Finally, it was assessed whether the T-score remained related to entropy after accounting for other factors that may influence image texture (age, sex, and BMI), using a multiple linear regression model (Table 3). The model was significant overall (*p* < 0.001), although it explained only a small proportion of the variability in entropy (R^2^ = 0.085; adjusted R^2^ = 0.071). The central finding was that, when the other factors were included, the T-score was no longer significantly associated with entropy (B = 0.0047; 95% CI: −0.001 to 0.011; *p* = 0.127). That is, the weak relationship observed did not hold as an effect specific to bone density once the other variables were considered. Age was also not associated with entropy (*p* = 0.661), and BMI showed only a weak positive relationship (B = 0.0018; *p* = 0.044). The only predictor with a clear and stable association was sex (B = 0.0332; *p* = 0.004); men showed higher entropy than women.

Inter-observer reproducibility of participant-level Shannon entropy was assessed in a random subsample of 30 participants selected from the primary analysis and independently reanalyzed by a second investigator. The analysis showed good inter-observer reliability, with an ICC of 0.862 (95% CI: 0.731–0.932). Mean entropy values were similar between Evaluator 1 (2.152) and Evaluator 2 (2.149), supporting the reproducibility of the entropy measurements across independent ROI delineations.

## 4. Discussion

This study assessed the relationship between the lumbar T-score and DXA-derived Shannon entropy in older adults. The relationship between the two variables was positive, statistically significant, and small in magnitude (r = 0.203), with approximately 4% shared variance. After adjustment for age, sex, and body mass index, the T-score was no longer significantly related to entropy, indicating that this metric did not behave as a direct reflection of BMD. Entropy values were virtually identical in participants with normal bone density and osteopenia, whereas the osteoporosis group showed significantly lower values and a moderate effect size. Likewise, nearly 28% of participants with normal density or osteopenia were in the lower entropy tertile, showing a discordance between densitometric classification and the image intensity distribution. Finally, sex was the variable that showed the most consistent relationship with entropy, with higher values in men.

The weak correlation between the T-score and entropy is consistent with the conceptual difference between the two measures. Areal BMD summarizes the amount of mineral projected over a given area, whereas Shannon entropy quantifies the diversity of gray levels within the analyzed region [11,12]. Some studies have shown that the texture of two-dimensional images is related to histomorphometric and three-dimensional parameters of trabecular bone, although these associations depend on the descriptor used and on the acquisition conditions [31]. In the clinical setting, Vokes et al. observed that texture analysis of calcaneal images generated by densitometry differentiated women with and without fractures and provided information complementary to BMD [32]. More recently, Liu et al. [33] summarized evidence showing that radiomic features derived from bone images can quantify geometric, intensity, and texture attributes linked to bone quality and strength. The review also noted that incorporating texture features into BMD may improve the estimation of bone strength compared with the use of BMD alone [33]. These antecedents support the interpretation that the low relationship observed in the present study does not necessarily indicate an absence of information, but rather that entropy may reflect texture variations not captured by the T-score. The Shannon entropy calculated in this study corresponds to a first-order radiomic feature derived from the intensity histogram; it quantifies the diversity of gray levels but does not incorporate their spatial distribution within the region of interest [20]. In contrast, DXA quantifies areal bone mineral density from a two-dimensional projection and expresses it through the T-score, without directly characterizing the spatial organization or the trabecular microarchitecture [9,10]. Therefore, the two metrics describe different and potentially complementary dimensions of bone tissue: the T-score mainly reflects the projected mineral quantity, whereas entropy characterizes the heterogeneity of the signal contained in the image.

In this context, the fact that the T-score did not maintain a statistically significant association with entropy after adjustment for age, sex, and BMI should not necessarily be interpreted as an unfavorable result. Since entropy is proposed as a complementary measure and not as a substitute for BMD, this low correspondence suggests that it does not redundantly reproduce the information contained in the T-score and that it could capture texture variations not reflected by the mineral quantity. However, the present study does not allow determining whether this additional information represents biological properties of bone tissue or technical factors related to image acquisition and processing, such as noise, segmentation, or quantization [33]. Therefore, its clinical utility will need to be confirmed through studies evaluating its relationship with microarchitecture, bone strength, and fracture risk.

The second finding was that entropy did not decrease gradually across the densitometric categories. Participants with normal bone density and osteopenia showed virtually identical values, whereas the reduction was observed only in the osteoporosis group. This behavior is not directly comparable with studies such as those by Rastegar et al. [21] and Cheng et al. [34] which succeeded in differentiating the densitometric categories using multivariate radiomic models. Unlike the present study, those models integrated multiple intensity and texture features, in addition to machine learning algorithms, whereas our analysis was limited to a single first-order feature. One possible explanation is that the alterations present in osteopenia are too subtle or depend on spatial patterns that cannot be captured by a descriptor based exclusively on the intensity histogram. In contrast, in osteoporosis, the changes in the distribution of gray levels could be sufficiently marked to produce a detectable decrease in entropy. This interpretation should be considered hypothetical, since lower entropy cannot be directly equated with greater deterioration of trabecular microarchitecture.

Along the same lines, the direction of the relationship between entropy and bone status has also not been uniform in studies conducted with other imaging modalities. De Castro Dytz et al. [31] observed higher vertebral entropy on computed tomography in patients with acromegaly, despite BMD being relatively preserved. In turn, Heilbronner et al. [22] reported that higher entropy calculated using co-occurrence matrices on magnetic resonance imaging was associated with lower volumetric BMD and with microarchitectural alterations assessed by high-resolution quantitative computed tomography. These differences do not necessarily represent contradictory results, since the studies differ in population, imaging modality, region analyzed, resolution, quantization, and mathematical definition of entropy [31]. In particular, entropy derived from co-occurrence matrices is a second-order feature that incorporates the spatial relationships between pixels, whereas the Shannon entropy used in this study is a first-order feature. Therefore, the meaning of high or low entropy values depends on the calculation method and the image context. Since no direct comparison with three-dimensional microarchitecture parameters was performed in the present study, entropy should be interpreted as a descriptor of DXA image texture and not as a direct measure of trabecular structure.

Another finding of the study was the discordance between densitometric classification and the entropy distribution. Of the participants with normal density, 27.5%, and of those with osteopenia, 29.0%, were in the lower entropy tertile, indicating that the two approaches do not necessarily identify the same individuals. This observation is consistent with the limited ability of the densitometric osteoporosis threshold to identify all individuals who will subsequently sustain a fracture. In the NORA study, Siris et al. [35] reported that 82% of the women who sustained a fracture during follow-up had a T-score above −2.5, although the individual fracture rate was higher among those meeting the densitometric criterion for osteoporosis. In addition, previous studies have shown that texture analysis of densitometric images can differentiate women with and without vertebral fracture, independently of BMD [32], whereas a radiomic score derived from hip DXA images provided additional capacity to predict hip fractures in older women [13]. However, in the present cohort the tertiles were defined from the sample distribution itself, and there are no clinical cutoff points for this measure. Therefore, belonging to the lower tertile should not be interpreted as synonymous with fragility, occult osteoporosis, or greater fracture risk; the finding identifies a phenotypic discordance that will need to be evaluated in longitudinal studies.

Another finding of this research was that, when the T-score, age, sex, and BMI were analyzed jointly using multiple linear regression, sex was the variable that showed the most consistent association with entropy. Sex differences have been documented in parameters of vertebral trabecular microarchitecture and in their age-related changes, which could contribute to distinct intensity patterns in the images [36]. However, the projected texture could also be influenced by body and technical characteristics related to acquisition. Therefore, the higher entropy observed in men cannot be directly interpreted as better trabecular organization.

Finally, from a clinical perspective, Shannon entropy should not be considered a substitute for the T-score or for established complementary DXA-derived measures such as the Trabecular Bone Score (TBS). TBS has an established role as an adjunct to BMD in fracture-risk assessment [11], whereas Shannon entropy represents a first-order radiomic feature within a broader quantitative imaging framework [20]. Therefore, the approach evaluated in the present study is not intended to compete with or replace TBS, but rather to explore whether an additional image-derived descriptor may provide complementary information from routinely acquired DXA images. Its main practical advantage is that it can be extracted from an already available DXA image without requiring a new acquisition or increasing radiation exposure. However, before proposing its use to reclassify patients or support clinical decisions, it will be necessary to demonstrate its reproducibility, stability against variations in acquisition and processing, comparability across devices, and additional value for predicting fractures beyond established measures. Although Hong et al. [13] showed that a multifeature radiomic score derived from hip DXA images improved fracture prediction beyond conventional clinical factors and T-score, those results cannot be directly extrapolated to a single first-order feature obtained from the lumbar spine.

Future studies should evaluate the reproducibility of entropy against variations in segmentation, acquisition, and processing; analyze the vertebrae separately; compare different quantization schemes; and incorporate second-order radiomic features. External validation in more diverse and sex-balanced cohorts will also be necessary, as will comparison with three-dimensional techniques such as vertebral quantitative computed tomography (QCT) or micro-computed tomography (micro-CT). Direct comparison with TBS should also be considered to determine whether radiomic features provide overlapping or complementary information to an established DXA-derived texture measure. Finally, longitudinal studies should determine whether entropy or more comprehensive radiomic signatures provide additional capacity to identify fractures or improve risk classification beyond BMD, clinical risk factors, and currently available complementary tools.

A central strength of this study was the assessment of the T-score and the radiomic feature from the same lumbar DXA image in each participant, which allowed direct comparison of densitometric and texture information without introducing differences arising from other imaging modalities, anatomical regions, or acquisition times. In addition, the sample included 271 older adults distributed across the three conventional densitometric categories, which made it possible to examine the behavior of entropy both along the continuous spectrum of the T-score and among participants with normal density, osteopenia, and osteoporosis. The internal consistency of the image analysis was supported by the use of a single densitometer calibrated daily, a uniform positioning and acquisition protocol, the anatomical delineation of the ROI within the vertebral body, and the assessment of inter-observer reproducibility in a randomly selected subsample. Another relevant aspect was the analytical strategy, which was not limited to comparing means between categories but integrated continuous associations, effect sizes, individual-level discordance analysis, and a multivariate model. This approach made it possible to assess not only whether entropy differed according to densitometric classification, but also to what extent it provided distinct and potentially complementary information to the T-score. Finally, the inclusion of functionally independent, community-dwelling older adults made it possible to explore this radiomic signal in a population not selected from osteoporosis clinics or fracture history, broadening its interest for future population-based applications.

This study also has several limitations. First, its cross-sectional design precludes establishing the temporal sequence of changes and, because prevalent or incident fractures were not included as an outcome, it was not possible to determine the clinical or prognostic value of entropy. Second, the sample was selected by convenience, was composed predominantly of women, and came from a single geographical region, which restricts the generalizability of the results. Third, DXA generates a two-dimensional image whose texture may be affected by soft-tissue thickness, noise, degenerative changes, and other projection artifacts. Although the most evident alterations were excluded, the residual influence of these factors cannot be ruled out. In addition, entropy depends on the size and delineation of the ROI, image resolution, intensity normalization, and the quantization scheme. Although inter-observer reproducibility was assessed in a subsample of participants, further studies in larger and independent samples are needed to confirm the reproducibility of the measure. Intra-observer reproducibility and test–retest stability were not assessed and should also be examined in future studies. Furthermore, TBS was not assessed in the present study, precluding direct evaluation of the relationship or potential complementarity between this established DXA-derived texture measure and Shannon entropy. Finally, the use of a single first-order radiomic feature allows description of the global distribution of gray levels but does not incorporate their spatial organization and, therefore, does not allow direct characterization of the orientation, connectivity, or arrangement of the trabeculae. Since no comparison with a three-dimensional reference technique was performed, entropy should be interpreted as a descriptor of DXA image texture and not as a direct measurement of vertebral microarchitecture.

## 5. Conclusions

This study showed that Shannon entropy derived from lumbar DXA images differs according to densitometric category and has only a weak unadjusted association with BMD, with no independent association with it after accounting for age, sex, and BMI. Participants with osteoporosis showed lower entropy values, whereas the groups with normal density and osteopenia showed similar values. In addition, a proportion of participants without densitometric osteoporosis were in the lower entropy tertile, evidencing a discordance between BMD-based classification and vertebral texture heterogeneity. These results suggest that entropy does not redundantly reproduce the information provided by the T-score and that it could provide a complementary characterization of DXA images. Nevertheless, its biological meaning and clinical utility cannot yet be established. For now, entropy should be considered an exploratory texture descriptor whose reproducibility should be further confirmed in larger and independent samples, and whose structural validity and ability to improve fracture prediction will need to be established in longitudinal and external validation studies.

## Figures and Tables

**Table 1:** Demographic, anthropometric, and clinical characteristics of the study population.

Variable	Total (n = 271)	Men (n = 50)	Women (n = 221)	*p* Value
Age (years)	72.5 ± 8.3 [71.5–73.4]	73.1 ± 6.9 [71.2–75.0]	72.0 ± 8.5 [70.9–73.1]	0.398
Weight (kg)	71.4 ± 13.9 [69.8–73.0]	81.5 ± 15.6 [77.1–85.8]	69.1 ± 12.3 [67.5–70.7]	<0.001
Height (m)	1.54 ± 0.09 [1.53–1.55]	1.66 ± 0.09 [1.63–1.68]	1.52 ± 0.06 [1.51–1.53]	<0.001
BMI (kg/m^2^)	29.8 ± 5.0 [29.2–30.4]	29.7 ± 4.8 [28.4–31.0]	29.8 ± 5.0 [29.1–30.5]	0.882
Waist circumference (cm)	99.4 ± 12.8 [97.9–100.8]	105.2 ± 11.9 [101.9–108.5]	97.8 ± 12.4 [96.1–99.5]	<0.001
T-score (DXA)	−1.58 ± 2.47 [−1.88–−1.29]	−0.34 ± 2.40 [−1.01–0.34]	−1.86 ± 2.40 [−2.18–−1.54]	<0.001
Entropy (bits)	2.141 ± 0.07 [2.13–2.15]	2.171 ± 0.07 [2.15–2.19]	2.134 ± 0.07 [2.13–2.14]	<0.001

Values are expressed as mean ± standard deviation and 95% confidence interval. BMI, body mass index; DXA, dual-energy X-ray absorptiometry; SD, standard deviation; CI, confidence interval.

**Table 2:** Profile of DXA image-texture heterogeneity (entropy) relative differences, and effect size by diagnostic category.

Diagnostic Group	T-Score Criterion	N	Entropy (Mean ± SD)	95% CI	Rel. Diff. (%)	Cohen’s d	*p*-Value †
Normal (Ref.)	≥−1.0	109	2.151 ± 0.063	2.139–2.163	—	—	—
Osteopenia	−2.5 < T < −1.0	93	2.150 ± 0.063	2.137–2.163	−0.05%	0.02	>0.99
Osteoporosis	≤−2.5	69	2.111 ± 0.076	2.093–2.129	−1.86%	0.59	<0.001

The Normal group is used as the reference for the calculation of relative differences and effect size. † *p*-values from the Tukey post hoc test versus the reference group.

**Table 3:** Multiple linear regression analysis for the predictors of Shannon entropy.

Predictor Variable	Coefficient (B)	Standard Error (SE)	t-Value	*p*-Value	95% CI
(Intercept)	2.1083	0.0535	39.42	<0.001	2.003 to 2.214
T-score	0.0047	0.0031	1.53	0.127	−0.001 to 0.011
Age (years)	−0.0003	0.0006	−0.44	0.661	−0.002 to 0.001
BMI (kg/m^2^)	0.0018	0.0009	2.03	0.044	0.000 to 0.004
Sex (Male)	0.0332	0.0114	2.92	0.004	0.011 to 0.056

n = 271; R^2^ = 0.085; adjusted R^2^ = 0.071; F(4, 266) = 6.18; *p* < 0.001. The reference category for the sex variable was female. BMI: body mass index; SE: standard error; CI: confidence interval.

## Data Availability

The data that support the findings of this study are available from the corresponding author upon reasonable request.

## Abbreviations

The following abbreviations are used in this manuscript:

BMD	Bone mineral density
BMI	Body mass index
DXA	Dual-energy X-ray absorptiometry
micro-CT	Micro-computed tomography
ROI	Region of interest
